# Sleep disturbances and quality of life in postoperative management after esophagectomy for esophageal cancer

**DOI:** 10.1186/1477-7819-12-156

**Published:** 2014-05-21

**Authors:** Marco Scarpa, Eleonora Pinto, Luca M Saadeh, Matteo Parotto, Anna Da Roit, Elisa Pizzolato, Rita Alfieri, Matteo Cagol, Elisabetta Saraceni, Fabio Baratto, Carlo Castoro

**Affiliations:** 1 Surgical Oncology Unit, Veneto Institute of Oncology (IOV-IRCCS), via Gattamelata 64, 35128 Padova, Italy; 2 ISTAR1 Intensive Care Unit, University of Padova, via Giustiniani 2, 35128 Padova, Italy

**Keywords:** Esophageal cancer, Esophagectomy, Postoperative management, Sleep disturbance

## Abstract

**Background:**

The aims of this prospective study were to analyze the predictors of postoperative sleep disturbance after esophagectomy for cancer and to identify patients at risk for postoperative hypnotic administration.

**Methods:**

Sixty two consecutive patients who underwent cancer-related esophagectomy were enrolled in this study from May 2011 to February 2012. Data about perioperative management, postoperative complications, ICU stay, and vasopressor, hypnotic, and painkiller administration were retrieved. The EORTC QLQ-C30 was used and global quality of life (QL2 item) and sleep disturbance (SL item) were the primary endpoints. Univariate and multivariate analyses were performed.

**Results:**

Postoperative request of hypnotics independently predicted bad quality of life outcome. Sleep disturbance after esophagectomy was independently predicted by the duration of dopamine infusion in the ICU and the daily request of benzodiazepines. Even in this case, only sleep disturbance at diagnosis revealed to be an independent predictor of hypnotic administration need. ROC curve analysis showed that sleep disturbance at diagnosis was a good predictor of benzodiazepine request (AUC = 73%, *P* = 0.02).

**Conclusions:**

The use of vasopressors in the ICU affects sleep in the following postoperative period and the use of hypnotics is neither completely successful nor lacking in possible consequences. Sleep disturbance at diagnosis can successfully predict patients who can develop sleep disturbance during the postoperative period.

## Background

Esophageal cancer is an increasingly common cancer with a poor prognosis. Its incidence has risen steadily over recent decades, and it is now the fastest rising solid tumor in most Western countries [1]. Nowadays, combined modality treatment protocols, such as neoadjuvant radiation and/or chemotherapy followed by esophagectomy, are the standard treatment since meta-analyses of randomized trials have found some survival advantages [2], especially in patients with a complete pathologic response to neoadjuvant therapy [3]. In a very recent and authoritative randomized controlled study, preoperative chemoradiotherapy was shown to improve survival among patients with potentially curable esophageal or esophagogastric-junction cancer [4]. Nevertheless, in spite of a limited (25% to 35%) possibility of cure and its association with a high risk of serious complications [5], esophagectomy remains part of the standard treatment for patients presenting with resectable esophageal cancer [6].

Postoperative management of patients undergoing esophagectomy is particularly challenging, requiring special expertise that can be found mainly in high volume centers [7]. In fact, the risk of severe postoperative complications is high even in specialized centers [6]; moreover, postoperative pain can heavily affect postoperative quality of life [8]. Sleep disruption by painful stimuli is frequently observed both in clinical and experimental conditions [9]. Furthermore, in spite of recent evidence showing that an early removal does not affect anastomotic outcome, a nasogastric tube is usually kept in place for the first 7 to 10 postoperative days causing constant discomfort [10]. Finally, after esophagectomy patients usually spend at least 2 days in the ICU, where noise and full light are almost constant throughout the day. A recent study showed that a range of hospital sounds have a high disruptive capacity on sleep, influencing both cortical brain activity and cardiovascular function [11]. All these premises suggest that patients undergoing esophagectomy would need hypnotic drugs to cope with postoperative sleep disturbances.

In a recent systematic review, we observed that, in the early postoperative period following esophagectomy, patients experience a significantly worsened global quality of life and are affected by more fatigue [12]. Moreover, we observed that postoperative pain, and its relief, are the main predictors of early postoperative quality of life after esophagectomy [8]. Therefore, the aims of this prospective study were to analyze the predictors of postoperative sleep disturbance after esophagectomy for cancer and to identify patients at risk for postoperative hypnotic administration.

## Methods

### Study design

**Figure 1:**
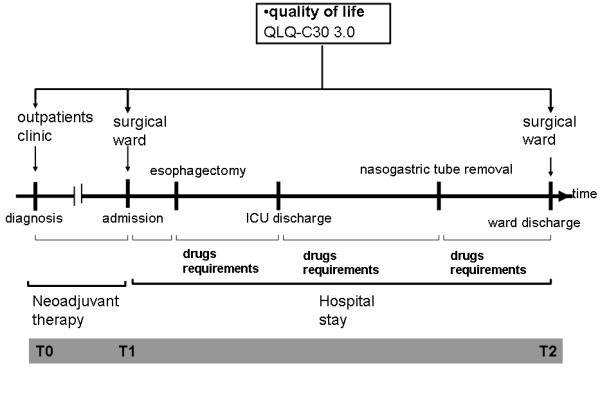
Study design.

Data from a prospectively collected database including all consecutive patients presenting with esophageal cancer at a tertiary referral center (the Surgical Oncology Unit of the Veneto Institute of Oncology, Padova, Italy) between May 2011 and September 2012, were reviewed. Clinical and socio-demographic data including age, sex, the type and timing of procedures carried out, and pre- and postoperative drug administration were prospectively recorded. The Clavien-Dindo classification of surgical complications was adopted for the classification of adverse events after surgery. Tumor-node-metastasis (TNM) staging was performed according to the most recent criteria of the International Union Against Cancer. Details concerning neoadjuvant therapy and surgical techniques have been published elsewhere [13]. The EORTC QLQ-C30, a measure assessing the quality of life of cancer patients, was administered to patients presenting to our outpatient clinic following neoadjuvant therapy at hospital admission for surgery and at hospital discharge. The study was performed according to the principles of the Declaration of Helsinki and all the patients gave their informed consent to data collection and study participation. The study was approved by the Ethical Committee of the Veneto Institute of Oncology (IOV-IRRCS) (internal code 2012/46). The study design is outlined in Figure 1.

### Administration of drugs influencing sleep during the postoperative period

During ICU stay, pain killers, inotropics, peridural anesthetic infusion, and hypnotic drug administration were monitored and quantified. Standard pain control protocol included naropine 0.2% epidural infusion (5 mL/h) and acetaminophen 1 g i.v. tris in die. Opioids (tramadol 100 mg or morphine 5 mg) were administered if pain persisted and, thus, upon the patients’ request. Benzodiazepines (lorazepam 2.5 mg, diazepam 10 mg, or lormetazepam 0.5 mg) were offered for insomnia to every patient and administered upon the patients’ request. The administration of pain killers or hypnotics upon request was registered on the clinical records by the nurse in charge. The daily dose requested was calculated.

### Quality of life questionnaire

The EORTC QLQ-C30 is a 30-item integrated system for assessing the generic quality of life of cancer patients [14]. The validity and robustness of the Italian version of the EORTC QLQ-C30 were assessed in a large series of cancer patients in 1998 [15]. It is grouped into five functional subscales (role, physical, cognitive, emotional, and social functioning) and two questions assessing overall quality of life. In addition, there are multi-item symptom scales (sleep, fatigue, pain, and nausea and vomiting). All of the scales and single-item measures range in score from 0 to 100. A high score for a functional scale represents a high/healthy level of functioning, a high score for the global health status/quality of life represents a high quality of life, and a high score for a symptom scale/item represents a high level of symptomatology/problems. The questionnaire has been validated into the Italian language.

### Statistical analysis

All statistical analyses were performed using the statistic program STATISTICA 5.1 for Windows 7 (Statsoft Inc.). The scores on the EORTC questionnaires were calculated according to the standard Quality of Life Group guidelines. The mean (95% CI) or frequency (%) was used for descriptive statistics unless otherwise described. Comparisons and correlations were carried out with Friedman ANOVA for paired multiple comparisons, Mann–Whitney U-test for unpaired comparison, and Kendall correlation test, respectively. Multiple regression models were created with significant predictors to determine the independent contributions of the different item scores. Receiving operator characteristics (ROC) were assessed by curve analysis. All tests were two-sided and a *P* value less than 0.05 was considered significant.

## Results

### Administration of drugs influencing sleep during the postoperative period

**Figure 2:**
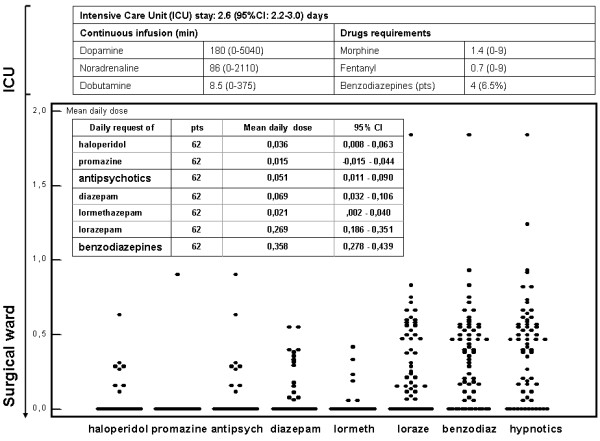
Administration of drugs influencing sleep during the postoperative period.

The patient, treatment, and cancer characteristics are outlined in Table 1. The median ICU stay was 2 (1–8) days long and the median epidural catheter stay was 5.2 (0–13) days long. The median infusion length was 180 (0–5,040) minutes for dopamine, 86 (0–2,110) for noradrenaline, and 8.5 (0–375) minutes for dobutamine. Four patients required benzodiazepines during the ICU stay. Once returned in the surgical ward, the mean daily request of diazepam, lormetazepam, and lorazepam was 0.069 (95% CI: 0.032–0.106), 0.021 (95% CI 0.002–0.040), and 0.269 (95% CI 0.186–0.351) doses, respectively. The overall benzodiazepine daily need was 0.358 (95% CI 0.278–0.439). Administration of drugs influencing sleep during the postoperative period is shown in Figure 2.

**Table 1:** Patient and cancer characteristics

**Patient characteristics**		
Demographics	Gender	13 F/49 M
	Age (years)	60 (27–84)
Symptoms at diagnosis	Weight loss (kg)	4.5 (0–22)
	Dysphonia (pts)	7 (11.3%)
	Pain (pts)	33 (53.2%)
	Burning (pts)	11 (17.7%)
	Reflux (pts)	18 (29.0%)
Comorbidities	Cardiologic comorbidities	32 (51.6%)
	Pulmonary comorbidities	14 (22.6%)
	Hepatic comorbidities	3 (4.8%)
	Psychiatric comorbidities	2 (3.2%)
**Cancer characteristics**		
Cancer site	Upper esophagus (pts)	2 (3.2%)
	Medium esophagus (pts)	8 (12.9%)
	Lower esophagus (pts)	52 (83.9%)
Histotype	Adenocarcinoma (pts)	43 (69.4%)
	Squamous cell carcinoma (pts)	19 (30.6%)
Pathological stage		
pT (pts)	pN (pts)	pM (pts)
T0 12	N0 34	M0 59
T1 10	N1 12	M1 3
T2 9	N2 6	
T3 26	N3 8	
T4 5		
**Esophagectomy**		
Surgical details	Cervical anastomosis/thoracic anastomosis (pts)	14 (22.6%)/48 (77.4%)
	Laparoscopy (pts)/thoracoscopy (pts)	6 (9.7%)/2 (3.2%)
	Feeding jejunostomy (pts)	31 (50%)
	Performing time (min)	428 (210–695)
	Selective lung exclusion (when performed) (min)	166 (65–390)
Esophagectomy complications	Anastomotic leaks (pts)	3 (4.8%)
	Cardiologic complication (pts)	7 (11.3%)
	Pulmonary complications (pts)	13 (21.0%)
	Urinary complication (pts)	4 (6.5%)
	Recurrent nerve lesions (pts)	4 (6.5%)

### Quality of life during treatment for cancer-related esophagectomy

**Figure 3:**
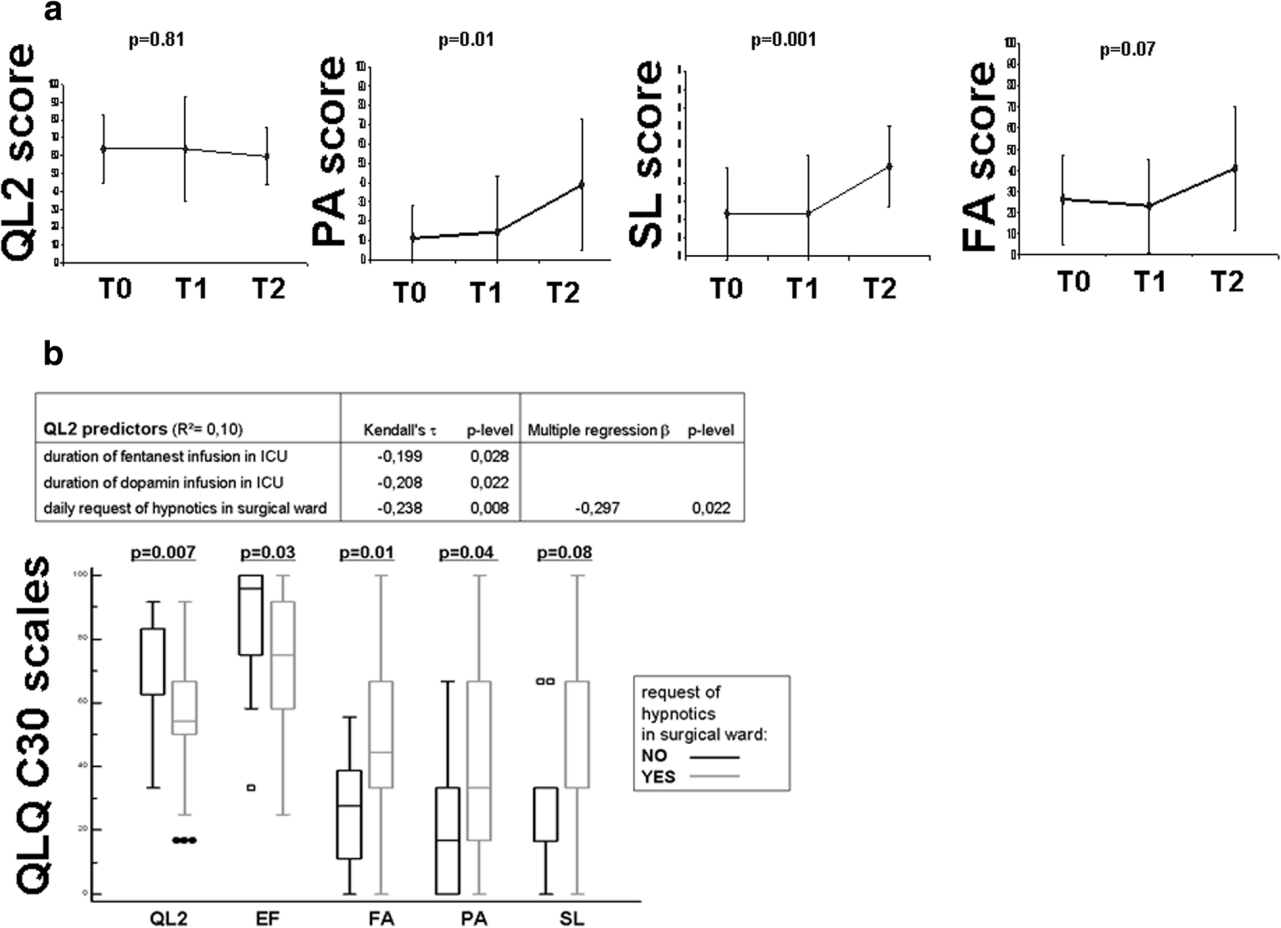
**Quality of life changes during the three steps of the study. (a)** Prospective assessment of QLQ C30 items (Friedman ANOVA). **(b)** Impact of postoperative treatment on quality of life after esophagectomy.

In our study group, the global quality of life and emotional functioning remained stable during the three steps of the study. On the contrary, pain and sleep disturbance significantly increased after surgery compared to after neoadjuvant therapy and at diagnosis (*P* = 0.01 and *P* = 0.001, respectively). Similarly, fatigue tended to increase after esophagectomy compared to after neoadjuvant therapy and at diagnosis (*P* = 0.07). The quality of life changes during the three steps of the study are shown in Figure 3a.

### Quality of life and request of drugs influencing sleep

In multivariate analysis, a daily request of hypnotics was an independent predictor of poor quality of life outcome (β = −0.297, *P* = 0.022). Emotional function after esophagectomy was independently predicted by the daily request of tramadol, the previous use of benzodiazepine, and the daily request of benzodiazepines (β = −0.354, *P* = 0.004; β = −0.335, *P* = 0.006; β = −0.242, *P* = 0.043, respectively). Fatigue after esophagectomy was independently predicted by sleep disturbance after neoadjuvant therapy and the daily request of benzodiazepines (β = 0.399, *P* = 0.008 and β = 0.288, *P* = 0.051, respectively). Sleep disturbance after esophagectomy was independently predicted by the duration of dopamine infusion in the ICU and the daily request of benzodiazepines (β = 0.236, *P* = 0.049 and β = 0.318, *P* = 0.010, respectively). The correlations between drugs influencing sleep administration and quality of life following surgery are outlined in Table 2.

**Table 2:** Predictors of global quality of life and of sleep disturbance

**Global quality of life after surgery predictors (R**^**2**^ **= 0.10)**	**Kendall’s τ**	***P *** **value**	**Multiple regression β**	***P *** **value**
Duration of fentanest infusion in ICU	−0.199	0.028	−0.148	0.244
Duration of dopamine infusion in ICU	−0.208	0.022		
Daily request of hypnotics in surgical ward	−0.238	0.008	−0.297	0.022
**Fatigue after surgery R**^**2**^ **= 0.28**	**Kendall’s τ**	***P *** **value**	**Multiple regression β**	***P *** **value**
Daily request of benzodiazepine in surgical ward	0.219	0.012	0.288	0.051
Sleep disturbance at admission	0.257	0.018	0.399	0.008
Postoperative cardiologic complications	0.186	0.033		
Duration of right lung exclusion	0.187	0.036		
**Emotional function after surgery R**^**2**^ **= 0.30**	**Kendall’s τ**	***P *** **value**	**Multiple regression β**	***P *** **value**
Daily request of tramadol in surgical ward	−0.211	0.019	−0.354	0.004
Previous use of benzodiazepine	−0.255	0.005	−0.335	0.006
Daily request of benzodiazepine in surgical ward	−0.275	0.002	−0.242	0.043
Duration of dopamine infusion in ICU	−0.205	0.024	−0.133	0.257
**Pain after surgery R**^**2**^ **= 0.24**	**Kendall’s τ**	***P *** **value**	**multiple regression β**	***P *** **value**
Cardiologic comorbidities	−0.304	0.001	−0.375	0.002
Maximum PEEP used in ICU	−0.224	0.016	−0.210	0.081
Duration of fentanest infusion in ICU	0.179	0.050	0.220	0.066
Daily request of benzodiazepine in surgical ward	0.187	0.040	0.157	0.189
**Sleep disturbance after surgery R**^**2**^ **= 0.23**	**Kendall’s τ**	***P *** **value**	**multiple regression β**	***P *** **value**
Daily request of benzodiazepine in surgical ward	0.286	0.002	0.318	0.010
Previous use of benzodiazepine	0.260	0.004	0.234	0.052
Emotional functioning after surgery	−0.197	0.030		
Duration of dopamine infusion in ICU	0.198	0.031	0.236	0.049
Psychiatric comorbidities	0.245	0.007		
Pain after surgery	0.198	0.031	0.231	0.057

Finally, patients who needed hypnotics during their postoperative period in the surgical ward had a significantly worse global quality of life compared to patients who did not need them (*P* = 0.007). Moreover, they reported a worse emotional function, fatigue, and pain (*P* = 0.03, *P* = 0.01, and *P* = 0.04, respectively). Curiously, in spite of the use of hypnotics, these patients tended to have a worse sleep disturbance (postoperative SL item) (*P* = 0.08). The effect of hypnotic administration on postoperative quality of life is shown in Figure 3b.

### Predictors of hypnotic administration in the postoperative period

**Figure 4:**
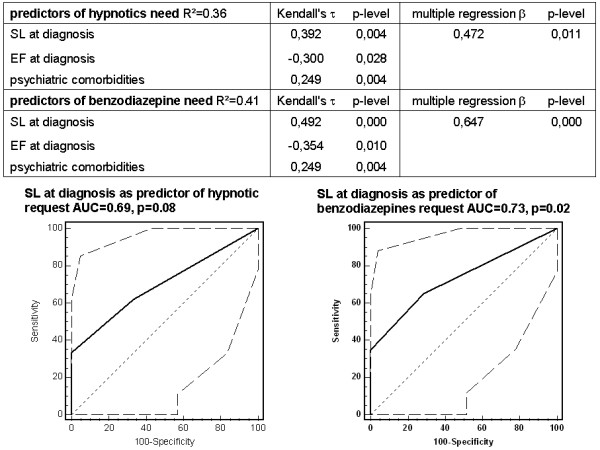
Predictors of request of hypnotics following esophagectomy.

In multivariate analysis, only sleep disturbance at diagnosis proved to be an independent predictor of postoperative request of any kind of hypnotic (β = 0.472, *P* = 0.011). ROC curve analysis showed that sleep disturbance at diagnosis was a relatively good predictor of postoperative request of any kind of hypnotic (area under the curve (AUC) of 69%, *P* = 0.08). Similarly, only sleep disturbance at diagnosis proved to be an independent predictor of postoperative benzodiazepine request (β = 0.647, *P* <0.001). ROC curve analysis showed that sleep distubance at diagnosis was a good predictor of postoperative benzodiazepines (AUC = 73%, *P* = 0.02). Predictors of hypnotic administration in the postoperative period are shown in Figure 4.

## Discussion

Postoperative management of patients undergoing esophagectomy is particularly challenging [7]. In fact, apart from the risk of severe postoperative complications, postoperative pain, fatigue, and sleep disturbances can heavily affect a patient’s postoperative quality of life [8]. Following esophagectomy, sleep disruption can occur because of painful stimuli [9], constant discomfort caused by the nasogastric tube [10], and the constant noise and light in the ICU. Moreover, in the long term, almost all post-esophagectomy patients experience some kind of reflux when supine and heartburn may make it difficult for patients to lie flat causing sleep disruption [16]. All these premises suggest that patients undergoing esophagectomy would require hypnotic drugs to cope with postoperative sleep disturbances. Therefore, the aim of this prospective study was to analyze how hypnotic and vasopressor administration affects postoperative sleep disturbance and quality of life following cancer-related esophagectomy.

In our series, patients spent, on average, 2 days in the ICU, where lights are often on for several hours per night. The role of the pineal gland is to translate light inputs from the retina into chemical signals for the rest of the body via production and secretion of melatonin to regulate the sleep/wake cycle. Melatonin production occurs on a night/day cycle and is heavily dependent on the concentration of serotonin [17,18]. Moreover, during ICU stay, patients often underwent inotrope and vasopressor infusion for cardiovascular support. The use of inotropes or vasopressors in the ICU has previously been correlated with post-discharge anxiety [19]. Intravenous dopamine does not pass the blood–brain barrier; however, unlike much of the rest of the mammalian brain, the pineal gland is not isolated from the body by the blood–brain barrier system and it has profuse blood flow, second only to the kidney. Recently, a new role for the D4 dopamine receptor in the pineal gland was observed: by means of a circadian-related heteromerization with adrenergic receptors their activation can limit the levels of melatonin secreted by the pineal gland [20]. The anatomic consideration and this new interesting observation might explain why, in our series, dopamine infusion in the ICU proved to be an independent predictor of sleep disturbance following esophagectomy.

In our series, sleep disturbance was significantly increased after surgery compared to baseline and it was independently predicted not only by the length of dopamine infusion but also, as expected, by daily request of benzodiazepines. Therefore, since in our series hypnotics were liberally administered upon the patients’ request after having reported insomnia, daily request of sedative hypnotics can be easily considered a marker of sleep disturbance. Four patients required benzodiazepines during ICU stay, and, once they returned to the surgical ward, the mean overall daily request of benzodiazepine was 35% of a dose and 40% for the hypnotic dose. However, in spite of the availability and the liberal use of hypnotics, these patients tended to experience worse sleep disturbances in the early postoperative period. Thus, the first take-home message is that, in the postoperative period, administration of hypnotics upon request does not completely fulfil its goal. Further strategies to manage postoperative insomnia following such a major surgery are warranted.

Moreover, it was already known that the administration of hypnotics, such as melatonin, following minimally invasive abdominal surgery, did not improve subjective sleep quality or well-being compared with placebo [21]. Even worse, in our observational series, we noted that patients requiring hypnotics in the surgical ward reported an impaired emotional function and global quality of life and worse fatigue and pain compared to patients who did not need them. Daily request of benzodiazepines in the postoperative period after esophagectomy was an independent predictor of impaired emotional function and increased fatigue. A strong association between depression at 3 months and receiving benzodiazepines in the ICU has been previously observed by Wade et al. [19]. However, the underlying mechanism is not clear since confounding factors might be associated with this phenomenon. Fatigue following esophagectomy was also predicted by sleep disturbance after neoadjuvant therapy and emotional function was also predicted by the daily request of tramadol; thus, sleep disturbances alone and postoperative pain may play a direct role on postoperative impairment of these quality of life aspects. Nevertheless, since daytime drowsiness and fatigue are well known short term side effects [22], and severe anxiety and depression and even suicide disturbances might be long term bad outcomes [23,24] of benzodiazepine use, the suspicion of a possible causative relation cannot be excluded. Therefore, although these findings should be interpreted cautiously given that hypnotics were not randomly assigned but were rather administered upon patient request, at clinician discretion, alternative treatments of postoperative insomnia should be initiated as first-line treatments in most patients [25].

These considerations make it necessary to identify patients at risk of sleep disturbance who might benefit from a preventive medical therapy or non-pharmacologic intervention [25]. In our series, postoperative daily hypnotics need and, in particular, daily benzodiazepine need in the surgical ward were independently predicted by the sleep disturbance item at diagnosis, although the accuracy of these predictions was not high but acceptable. This simple tool (a single question investigating the presence of sleep disturbance in the previous week) might be used to quickly screen patients for whom esophagectomy may be a therapeutic option. Once they are admitted for the operation they may have a better management of postoperative insomnia.

## Conclusions

In conclusion, the use of vasopressors in the ICU affects sleep in the subsequent postoperative period, and the use of hypnotics and, in particular, of benzodiazepines is neither completely successful nor lacking in possible consequences in terms of impaired emotional function and quality of life and worse postoperative fatigue and pain. The sleep disturbance item at diagnosis can successfully predict patients who can develop sleep disturbances during their postoperative period and can be used as a quick screening test to plan further interventions that might help reduce poor outcomes following esophagectomy.

## Competing interests

The authors declare that they have no competing interest to declare and that they have full control of all primary data and agree to allow the journal to review their data if requested.

## Authors’ contributions

MS and EP gave substantial contributions to conception and design, to acquisition of data and to analysis and interpretation of data and they were involved in drafting the manuscript. LMS and MP gave substantial contributions to acquisition of data and interpretation of data and he was involved in critical revising for important intellectual content. ADR, EP, RA, MC, ES, and FB gave substantial contributions to acquisition of data and they were involved in critical revising for important intellectual content. CC gave substantial contributions to conception and design and interpretation of data and he was involved in drafting the manuscript. All authors read and approved the final manuscript.
